# Implantation of dedifferentiated fat cells ameliorated antineutrophil cytoplasmic antibody glomerulonephritis by immunosuppression and increases in tumor necrosis factor-stimulated gene-6

**DOI:** 10.1186/s13287-022-03014-8

**Published:** 2022-07-16

**Authors:** Kei Utsunomiya, Takashi Maruyama, Satoshi Shimizu, Taro Matsumoto, Morito Endo, Hiroki Kobayashi, Koichiro Kano, Masanori Abe, Noboru Fukuda

**Affiliations:** 1 Division of Nephrology Hypertension and Endocrinology, Department of Medicine, Nihon University School of Medicine, Tokyo, Japan; 2 Division of Cell Regeneration and Transplantation, Department of Functional Morphology, Nihon University School of Medicine, Tokyo, Japan; 3 Faculty of Human Health Science, Hachinohe Gakuin University, Hachinohe, Aomori, Japan; 4 Laboratory of Cell and Tissue Biology, College of Bioresource Science, Nihon University, Fujisawa, Japan

**Keywords:** ANCA glomerulonephritis, Implantation, DFAT, TSG-6, Macrophage

## Abstract

**Introduction:**

The implantation of dedifferentiated fat (DFAT) cells has been shown to exert immunosuppressive effects. To develop DFAT cell therapy for antineutrophil cytoplasmic antibody (ANCA) glomerulonephritis, the effects of the implantation of DFAT cells on ANCA glomerulonephritis were investigated in mice.

**Methods:**

PKH26-labeled DFAT cells (10^5^) were infused through the posterior orbital venous plexus to investigate delivery of DFAT cells in ICR mice. DFAT cells (10^5^) were also implanted in SCG mice as a model for ANCA glomerulonephritis. Expression of tumor necrosis factor-stimulated gene-6 (TSG-6) mRNA and protein in kidney was evaluated, and the expression of microRNAs associated with TSG-6 in plasma, lung and kidney was analyzed. Expressions of CD44, prostaglandin (PG) E2, interleukin (IL)-10, IL-1*β*, tumor necrosis factor (TNF)-*α* mRNAs, C–C motif chemokine ligand 17 (CCL-17) and monocyte chemoattractant protein (MCP)-1 proteins were measured in kidney from SCG mice implanted with DFAT cells.

**Results:**

After their intravenous infusion, almost all DFAT cells were trapped in the lung and not delivered into the kidney. Implantation of DFAT cells in SCG mice suppressed glomerular crescent formation, decreased urinary protein excretions and increased expression of TSG-6 mRNA, protein and immunostaining in kidney from these mice. Increased expression of microRNA 23b-3p in plasma, kidney and lung; decreased expression of CD44 mRNA; and increased expression of PGE2 and IL-10 mRNAs were also observed in kidney from these mice. Implantation of DFAT cells also decreased the expression of TNF-*α* and MCP-1 proteins and increased that of CCL-17 protein in kidney from the SCG mice. Survival rates were higher in SCG mice implanted with DFAT cells than in SCG mice without implantation.

**Conclusion:**

Mechanisms underlying the effects of improvement of ANCA glomerulonephritis are associated with immunosuppressive effects by TSG-6 and the transition of M1–M2 macrophages, suggesting that implantation of DFAT cells may become a cell therapy for ANCA glomerulonephritis.

**Supplementary Information:**

The online version contains supplementary material available at 10.1186/s13287-022-03014-8.

## Introduction

Implantation of mesenchymal stem cells (MSCs) has recently been reported to repair tissue injuries through anti-inflammatory and immunosuppressive effects [1]. It has been established that dedifferentiated fat (DFAT) cells show identical characteristics to MSCs [2, 3]. Immunosuppressive effects of MSCs have been reported to be mediated by MSC-released immune modulators such as indoleamine 2,3-dioxygenase [4], prostaglandin E2 (PGE2) [5], inducible nitric oxide synthase [6], transforming growth factor *β* [7], interleukin (IL)-10 [8], hepatocyte growth factor [9] and tumor necrosis factor-stimulated gene-6 (TSG6) [10]. Systematic infusion of MSCs has also been reported to suppress graft rejection in animal models [11], and the implantation of MSCs investigated in clinical studies has been reported to effectively inhibit graft-versus-host disease [12]. As one method of implanting MSCs, the systematic implantation of DFAT cells effectively ameliorated antibody-induced glomerulonephritis through immunosuppressive effects accompanied by the suppression of macrophage infiltration, and it increased the production of serum and renal TSG-6, which improved antibody-induced renal degeneration. These findings suggest that DFAT cells can potentially be a suitable cell source for the treatment of immunological progressive renal diseases [13]. Systematic implantation of DFAT cells effectively ameliorated monoclonal antibody (mAb) 1-22-3-induced glomerulonephritis through immunosuppressive effects accompanied by the suppression of macrophage infiltration and the expression of IL-6, IL-10 and IL-12*β* , and increased the production of serum and renal TSG-6, which improved the mAb 1-22-3-induced renal degeneration, through its immunosuppressive effects alone. Thus, DFAT cells may be a suitable cell source for the treatment of immunological progressive renal diseases.

Autoimmune-associated kidney diseases such as antineutrophil cytoplasmic antibody (ANCA) glomerulonephritis and lupus nephritis have been refractory diseases in the clinical field. A typical renal pathological finding is that of glomerular necrotic crescent formation. Mild lesions of ANCA glomerulonephritis show segmental necrotic glomerulonephritis, and most severe lesions show glomerular necrotic crescent formation [14]. As a model for ANCA glomerulonephritis, the spontaneous crescentic glomerulonephritis-forming (SCG) mouse is a hybrid inbred strain established by brother–sister inbreeding of the BXSB mouse to induce crescent-forming glomerulonephritis and the MRL/lpr mouse to induce ANCA-associated vasculitis. Thus, the SCG mouse is a genetic model mouse with the autoimmune promoting gene lpr [15].

ANCA-associated vasculitis induces pauci-immune necrotizing and crescentic glomerulonephritis. ANCA glomerulonephritis shows acute lesion in glomeruli and other vessels in the kidney with vessel wall necrosis that releases constituents of the plasma, including coagulation factors, into the necrotic zone. ANCAs induce activation of neutrophils to attack small vessels. ANCA glomerulonephritis is the most frequent disease leading to rapidly progressive glomerulonephritis, and its life prognosis is poor because repeat relapses of this disease occur after transient improvement with steroid therapy [16]. The number of patients with rapidly progressive glomerulonephritis as a cause of dialysis-introduced primary disease has recently begun to swiftly rise in Japan. Radical therapies are therefore required to rescue patients with ANCA glomerulonephritis. Cell-based therapies with DFAT cells are expected as one form of radical therapy. A limitation of DFAT cell therapy is the lack of knowledge on the mechanisms underlying its immunosuppressive effects.

The aim of this study was to develop DFAT cell therapy for ANCA glomerulonephritis. The effects of the implantation of DFAT cells on renal function, proteinuria and glomerulonephritis in SCG mice were examined as a preclinical study of DFAT cell therapy for ANCA glomerulonephritis, and mechanisms of the immunosuppressive effects of the implantation of DFAT cells were also investigated.

## Methods

### Ethics and animals

This investigation conformed to the Guide for the Care and Use of Laboratory Animals published by the US National Institutes of Health (NIH Publication No. 85-23, 1996). The ethics committee of the Nihon University School of Medicine approved this study (approval no.: AP18MED021-1). Male SCG/Kj mice were purchased from the National Institutes of Biomedical Innovation, Health and Nutrition and were bred with female ICR mice (Charles River Laboratory Japan, Yokohama, Japan).

### Preparation of DFAT cells

Preparation of DFAT cells from mouse adipose tissue was performed as described previously [3]. Briefly, around 1 g of epididymal adipose tissue from six male ICR mice was treated with collagenase and centrifuged. Adipocytes were isolated from the top layer. More than 99% of the isolated cells were confirmed to be mature lipid-filled adipocytes by staining with AdipoRed (Cambrex, Walkersville, MD, USA). The mature adipocytes floating on top of the culture medium attached to the upper surface of the culture flasks within a few days. Approximately 10–20% of the adherent cells flattened out by day 3 and changed into a spindle-shaped morphology by day 7. The cells subsequently entered a proliferative log-phase upon inversion of the flasks and changing of the media and reached confluence by day 14. During this stage, the cells completely lose their lipid droplets and exhibit the fibroblast-like morphology of DFAT cells. The DFAT cells from the six different ICR mice were harvested and combined as allogenic DFAT cells.

### Distribution of DFAT cells after implantation

**Fig. 1:**
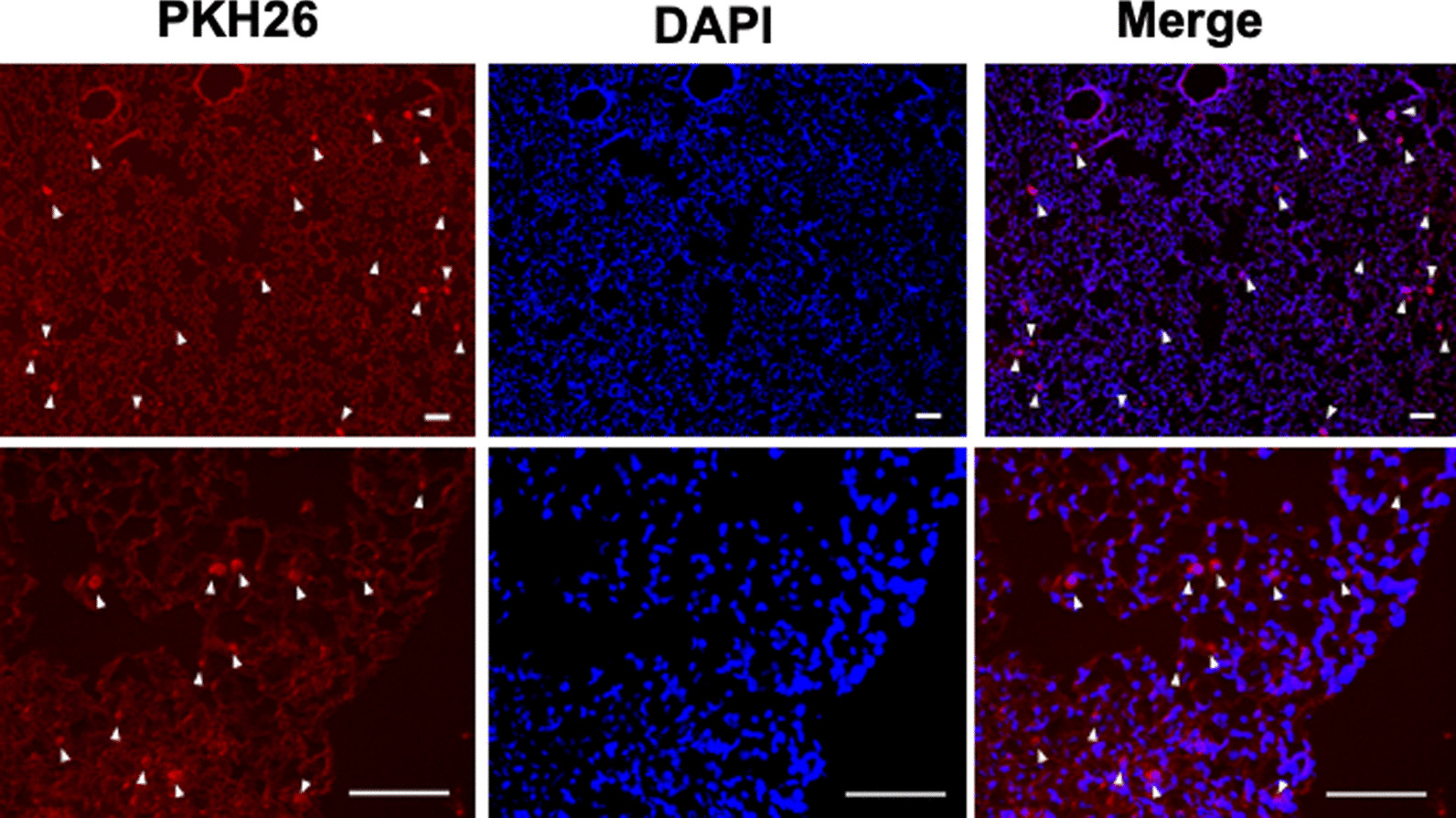
Distribution of implanted DFAT cells. In total, 10^5^ PKH26-labeled DFAT cells were infused through the posterior orbital venous plexus in ICR mice. One hour, 24 h, and one week after the infusion, kidney, aorta, liver and lungs were removed and fixed in 3% formalin in PBS and embedded in paraffin. Arrowheads indicate trapped DFAT cells. Bar = 50 μm. DFAT dedifferentiated fat

DFAT cells from the ICR mice were labeled with PKH26 Red Fluorescent Cell Linker Kit for General Cell Membrane Labeling (Sigma Chemical, St. Louis, MO, USA) as described previously [13]. In total, 10^5^ labeled DFAT cells were infused through the posterior orbital venous plexus of the ICR mice. At 1 h, 24 h and 1 week after the injection, kidney, aorta, liver and lungs were removed and fixed in 3% formalin in phosphate-buffered saline (PBS) (Kanto Chemical, Tokyo, Japan) and embedded in paraffin. Sections were observed under a fluorescence microscope (IX73; Olympus, Tokyo, Japan), and images were obtained with a digital imaging system (Fig. 1).

### Experimental protocols

**Fig. 2:**
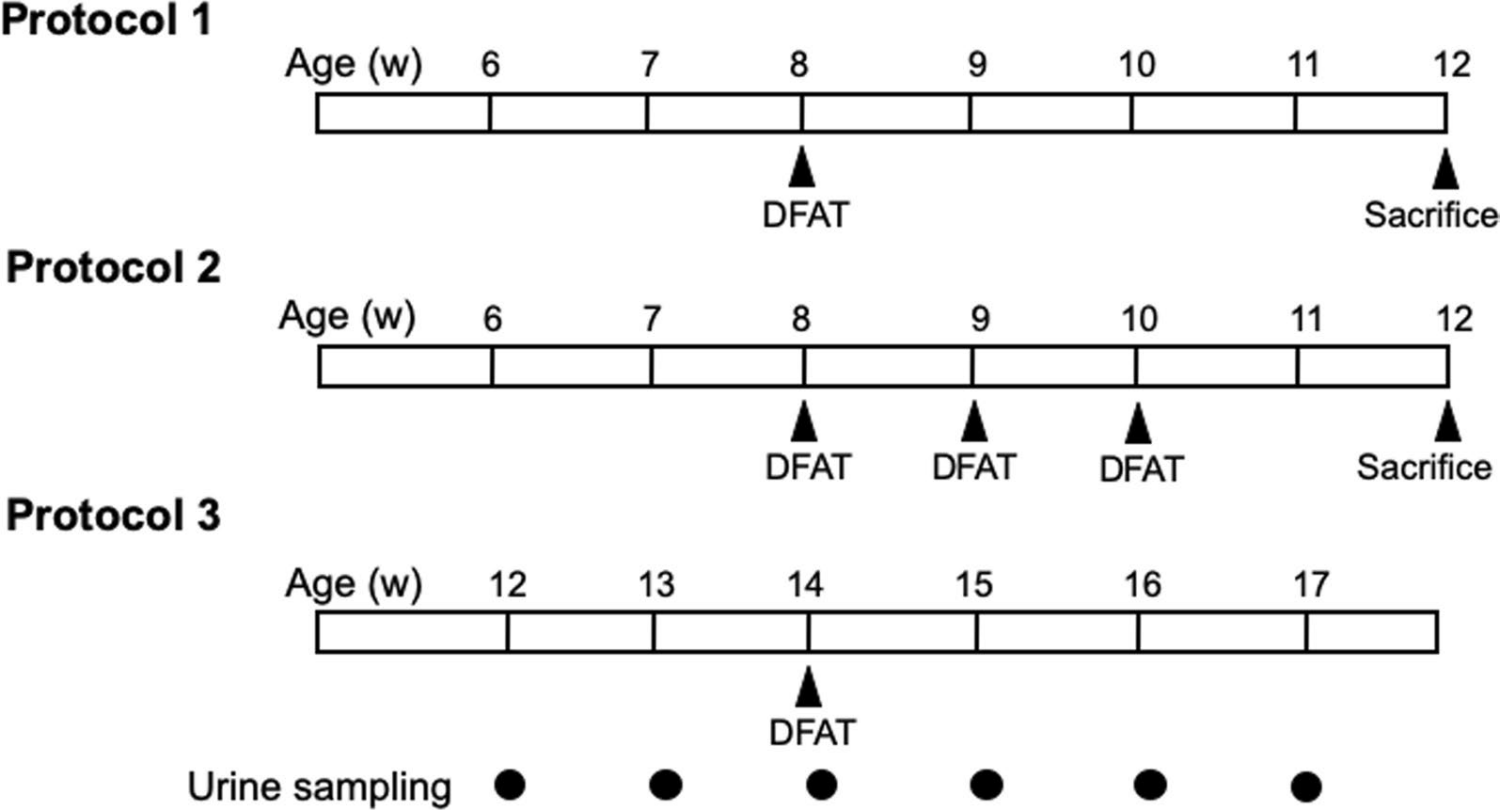
Experimental protocols for the implantations of DFAT cells in SCG mice. Protocol 1: SCG mice with single implantation of DFAT cells at 8 weeks of age. Protocol 2: SCG mice with respective triple implantation of DFAT cells at 8, 9 and 10 weeks of age. Each mouse was killed at 12 weeks of age, its blood sampled, and its kidney removed. Protocol 3: Excreted urinary protein was collected from SCG mice weekly from 12 to 17 weeks of age in metabolic cages. DFAT dedifferentiated fat

Figure 2 shows the protocols used for the implantations of DFAT cells. ICR mice without implantation of DFAT cells were the control for SCG mice, and SCG mice without implantation of DFAT cells were the control for the implantations of DFAT cells. Protocol 1 indicates SCG mice with single implantation of 10^5^ DFAT cells at 8 weeks of age. Protocol 2 indicates SCG mice with respective triple implantation of 10^5^ DFAT cells at 8, 9 and 10 weeks of age. Each mouse was killed at 12 weeks of age, blood was sampled, and kidneys were removed. Protocol 3 indicates SCG mice with single implantation of DFAT cells at 8 weeks of age. Urine was collected from these mice weekly from 12 to 17 weeks of age in metabolic cages, and urinary protein was determined with a Bio-Rad protein assay kit (Bio-Rad, Hercules, CA, USA). Serum blood urea nitrogen (BUN) and creatinine were measured by SRL, Inc. (Wako, Saitama, Japan). The myeloperoxidase (MPO)-ANCA titer was determined by an enzyme-linked immunosorbent assay kit (MBL Laboratory, Tokyo, Japan).

### Determination of renal injury

The 3-mm paraffin-embedded sections of removed renal cortex were stained with hematoxylin and eosin. Renal cortical thickness was measured under high magnification (× 400). The glomerular injury score (GIS) was obtained by the following formula: [(0 × *n*0) + (1 × *n*1) + (2 × *n*2) + (3 × *n*3) + (4 × *n*4)]/50. To semi-quantify the tubulointerstitial area, 20 areas of the renal cortex were randomly selected. The percentage of each area that showed sclerofibrotic change was estimated and assigned a score of 0, normal; 1, involvement of < 10% of the area; 2, involvement of 10–30% of the area; 3, involvement of > 30–50% of the area; or 4, involvement of > 50% of the area. The tubulointerstitial injury score (TIS) was similarly calculated as [(0 × *n*0) + (1 × *n*1) + (2 × *n*2) + (3 × *n*3) + (4 × *n*4)]/20.

### Immunohistochemical analysis of TSG-6

For immunohistochemical analysis of TSG-6, rabbit polyclonal anti-TSG-6 antibody (Santa Cruz Biotechnology, Santa Cruz, CA, USA) diluted 1:500 was used as the first antibody and incubated for 30 min, followed by ImmPRESS Reagent (VECTOR LABORATORIES, Burlingame, CA, USA) as the secondary antibody. Counterstaining was then performed before the sections were examined under a light microscope.

### RNA extraction and real-time polymerase chain reaction (PCR)

Total RNA was extracted from renal medulla of kidney of 12-week-old mice with TRIzol reagent (Life Technologies) according to the manufacturer’s instructions. Total RNA (1 μg) was reverse transcribed into cDNA with random 9-mers with an RNA PCR Kit (AMV) Ver. 3.0 (Takara Bio, Ohtsu, Japan). With the use of a StepOnePlus real-time PCR system (Applied Biosystems, Bedford, MA, USA), mRNA expression of TSG-6 (Tnfaip6), CD44 (Cd44), PGE2 (Ptger2), IL-10 (Il10), IL-1*β* (Il1b), tumor necrosis factor (TNF)-*α* (Tnf) and *β*-actin (Actb) as an internal control was determined by the SYBR Green method with Power UP SYBR^®^ Green PCR Master Mix (Applied Biosystems).

Amplifications were done at 95 °C for 15 s, then 60 °C for 60 s and 95 °C for 15 s for 55 cycles with a GeneAmp PCR System 2700 (Applied Biosystems). After we determined the threshold cycle (Ct), we used the comparative Ct method to calculate the relative quantification of mRNA expression of the marker gene. The quality and concentration of the amplified PCR products were determined using an Agilent 2100 Bioanalyzer (Agilent, Palo Alto, CA, USA). Table 1 shows the sequences of these primers.

**Table 1:** Sequences of primers for real-time PCR

Tnfaip6	Forward 5′ to 3′	GCT GTC CTG GAA CTC ACT TTG
	Reverse 5′ to 3′	GAG GCA GGT GGA TTT CTG AG
Cd44	Forward 5′ to 3′	TCC TTC TTT ATC CGG AGC AC
	Reverse 5′ to 3′	CCT GGA GTC CTT GGA TGA GT
Ptger2	Forward 5′ to 3′	ATC ACC TTC GCC ATA TGC TC
	Reverse 5′ to 3′	GCT CGG AGG TCC CAC TTT
Tnf	Forward 5′ to 3′	TCT TCT CAT TCC TGC TTG TGG
	Reverse 5′ to 3′	GGT CTG GGC CAT AGA ACT GA
Il10	Forward 5′ to 3′	CAG AGC TCC TAA GAG AGT TGT GAA
	Reverse 5′ to 3′	TCA TCA AAG GAT CTC CCT GGT
Il1b	Forward 5′ to 3′	AGT TGA CGG ACC AAA G
	Reverse 5′ to 3′	AGC TGG ATG CTC TCA TCA GG
Actb	Forward 5′ to 3′	CCA ACC GTG AAA AGA TGA CC
	Reverse 5′ to 3′	ACC AGA GGC ATA CAG GGA CA

### Quantification of microRNA (miRNA)

Peripheral blood was collected by cardiac blood samplings from three SCG mice each with or without implantation of DFAT cells and mixed with an equal volume of PBE buffer solution (containing 10% fetal bovine serum, 2 mM ethylenediaminetetraacetic acid and PBS. Peripheral blood mononuclear cells were isolated by the Percoll method (Sigma-Aldrich, St. Louis, MO, USA, https://www.sigmaaldrich.com/JP/ja/technical-documents/protocol/cell-culture-and-cell-culture-analysis/mammalian-cell-culture/how-to-make-and-use-gradients-of-percoll). Kidneys and lungs from three SCG mice each with or without implantation of DFAT cells were homogenized and mixed in PBE buffer solution. Total RNA was isolated from the PBE-mixed samples from kidney and lung with TRIzol Reagent (Invitrogen, Waltham, MA, USA) according to the manufacturer’s protocol. Total RNA was purified and ultimately eluted into 20 μL of RNase-free water. RNA quantity was assessed with a NanoDrop system (NanoDrop Products, Wilmington, DL, USA).

Sequencing libraries were constructed using the QIAseq^™^ miRNA Library Kit (Qiagen, Hilden, Germany) according to the manufacturer’s protocols. The quality of the libraries was assessed with an Agilent Bioanalyzer using a High Sensitivity DNA chip (Agilent Technologies, Santa Clara, CA, USA). The pooled libraries of the samples were sequenced using NextSeq 500 (Illumina, Inc., San Diego, CA, USA) in 76-base pair single-end reads.

The QIAseq miRNA library kit adopts the Unique Molecular Indexes (UMI) system, enabling unbiased and accurate quantification of mature miRNAs. Original FASTQ files generated by NextSeq were uploaded to the Qiagen GeneGlobe Data Analysis Center (https://geneglobe.qiagen.com) and aligned to the miRBase v21 (http://www.mirbase.org) and piRNABank (http://pirnabank.ibab.ac.in/). All reads assigned to a particular miRNA or piRNA were counted, and the associated UMI were aggregated to count unique molecules. A matrix of the UMI counts of miRNA or piRNA was subjected to downstream analyses using StrandNGS 3.4 software (Agilent Technologies). The UMI counts were quantified using a trimmed mean of M-value method [17]. miRNAs were annotated against miRBase.

### Western blot analysis

Renal medulla from mice were disrupted with lysis buffer (50 mM Tris·HCl, pH 8.0, 150 mM NaCl, 0.02% sodium azide, 100 μg/mL phenylmethylsulfonyl fluoride, 1 μg/mL aprotinin and 1% Triton X-100). Total proteins were extracted and purified with 100 μL of chloroform and 400 μL of methanol. Protein samples were boiled for 3 min and subjected to electrophoresis on 8% polyacrylamide gels and then transblotted to nitrocellulose membranes (Bio-Rad Laboratories). Blots were incubated with anti-TSG-6 polyclonal antibody (Santa Cruz Biotechnology), anti-TNF-*α* polyclonal antibody (Cell Signaling Technology, Danvers, MA, USA), anti-monocyte chemoattractant protein-1 (MCP-1) polyclonal antibody (Biorbyt, Cambridge, UK), anti-C–C motif chemokine ligand 17 (CCL-17) polyclonal antibody (Invitrogen) and anti-beta actin antibody (Abcam, Cambridge, UK) in 5% non-fat milk in tris-buffered saline with 0.1% Tween 20 detergent (TBST) solution (10 mM Tris·HCl, pH 8.0, 150 mM NaCl and 0.05% Tween 20) for 3 h at room temperature. The membrane was incubated with horseradish peroxidase-labeled secondary antibody (Sigma-Aldrich) for 1 h at room temperature and then washed with TBST once for 15 min and then four more times for 5 min each. Immune complexes on the membrane were detected by the enhanced chemiluminescence method (Amersham Pharmacia Biotech, Little Chalfont, Buckinghamshire, UK). Obtained bands were quantified by ImageStudio Lite (https://www.licor.com/bio/image-studio-lite).

### Determination of regulatory T cell population

Spleen was removed and homogenized 4 weeks after implantation of 10^6^ DFAT cells in SCG mice. Spleen cell suspensions were centrifuged at 1.5 × 10^3^ rpm to collect the cells in 100 μL of flow cytometry staining buffer. Anti-mouse cluster of differentiation-4 (CD4) (CD4-APC-Cy7, Abcam) and anti-mouse CD25 (CD25-AlexaFluor 647, BioLegend, San Diego, CA, USA) were added to each buffer and incubated in the dark for 30 min at 4 °C. After surface antibody labeling, the cells were washed twice with flow cytometry staining buffer before fixation/permeabilization working solution (1 mL) was added to resuspend the cells, and before they were incubated overnight in the dark at 4 °C. Anti-mouse Foxp3 antibody (FOXP3-PerCP5.5, BD Biosciences, San Jose, CA, USA) was then added, and the cells were again incubated in the dark for 30 min at 4 °C to evaluate the proportion of CD4^+^ CD25^+^ FOXP3^+^ regulatory T cells present. Cells were fixed and permeabilized with a FOXP3 Staining Buffer Set according to the manufacturer’s instructions (https://www.thermofisher.com/jp/ja/home/references/protocols/cell-and-tissue-analysis/protocols/staining-intracellular-antigens-flow-cytometry.html) and included a blocking step with 2% rat serum. Flow cytometry was performed with an Accuri C6 Flow Cytometer (BD Biosciences).

### Statistics

Values are reported as the mean ± SEM. Statistical analyses were performed using JMP 10 software (SAS, Tokyo, Japan), and statistical significance was calculated using two-way ANOVA with the Bonferroni/Dunn procedure used as a post-test. A value of *P* < 0.05 was considered to indicate statistical significance.

## Results

### Distribution of implanted DFAT cells

To evaluate distribution of the implanted DFAT cells, 10^5^ PKH26-labeled DFAT cells were infused through the posterior orbital venous plexus of ICR mice. One hour after injection, these DFAT cells were trapped in the lung (Fig. 1) and were not delivered into the kidney, aorta or liver. The PKH26-positive cells were trapped for one day and had disappeared from the lung by 1 week after implantation (data not shown).

### Effects of single versus triple implantations of DFAT cells on renal function, MPO-ANCA and TSG-6

**Fig. 3:**
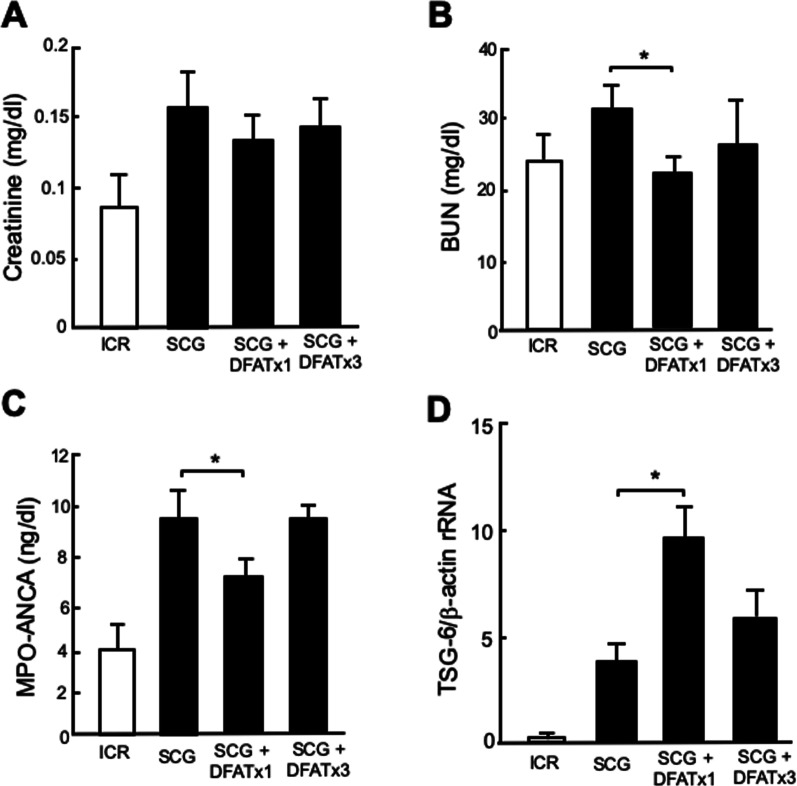
Comparison of single and triple implantations of DFAT cells for renal function, MPO-ANCA titer and expression of TSG-6 mRNA in kidney from SCG mice. SCG mice were infused with 10^5^ DFAT cells through the posterior orbital venous plexus once at 8 weeks of age (DFAT × 1) or three times at 8, 9 and 10 weeks of age (DFAT × 3). Each mouse was killed at 12 weeks of age, its blood sampled to determine creatinine (**A**), blood urea nitrogen (BUN) (**B**) and titer of myeloperoxidase-antineutrophil cytoplasmic antibody (MPO-ANCA) (**C**), and its kidney removed to determine expression of TSG-6 mRNA (**D**). Total RNA was extracted from renal cortex and medulla from kidney, and mRNA expression of TSG-6 was determined by a real-time PCR system. Data are shown as the mean ± SEM (*n* = 4). *P < 0.05 in the indicated columns. SEM standard error of the mean; TSG-6 tumor necrosis factor-stimulated gene-6

Effects of single and triple implantations of DFAT cells were then examined on renal function, plasma MPO-ANCA titer and expression of TSG-6 mRNA in kidney. Single and triple implantations of DFAT cells did not affect the increased serum creatinine levels in SCG mice (Fig. 3A). Single implantations of DFAT cells significantly (*P* < 0.05) suppressed increased BUN levels in SCG mice, but triple implantations of DFAT cells had no significant effect on BUN levels (Fig. 3B). Single implantation of DFAT cells significantly (*P* < 0.05) suppressed the increased plasma MPO-ANCA titer in SCG mice, whereas triple implantations of DFAT cells did not affect the plasma titer (Fig. 3C). Figure 3D shows the effects of single and triple implantations of DFAT cells on the expression of TSG-6 mRNA in kidney from SCG mice. Single implantations of DFAT cells significantly (*P* < 0.05) increased the amount of TSG-6 mRNA in kidney from SCG mice, whereas triple implantations of DFAT cells had no significant effect on TSG-6 mRNA expression. Thus, the single implantations of DFAT cells rather effectively improved renal function, decreased the MPO-ANCA titer, and increased the expression of TSG-6 in kidney in SCG mice compared with the effects from triple implantations of DFAT cells. Therefore, a single implantation of DFAT cells in SCG mice was used in the following experiments.

### Effects of implantation of DFAT cells on renal injury in SCG mice

**Fig. 4:**
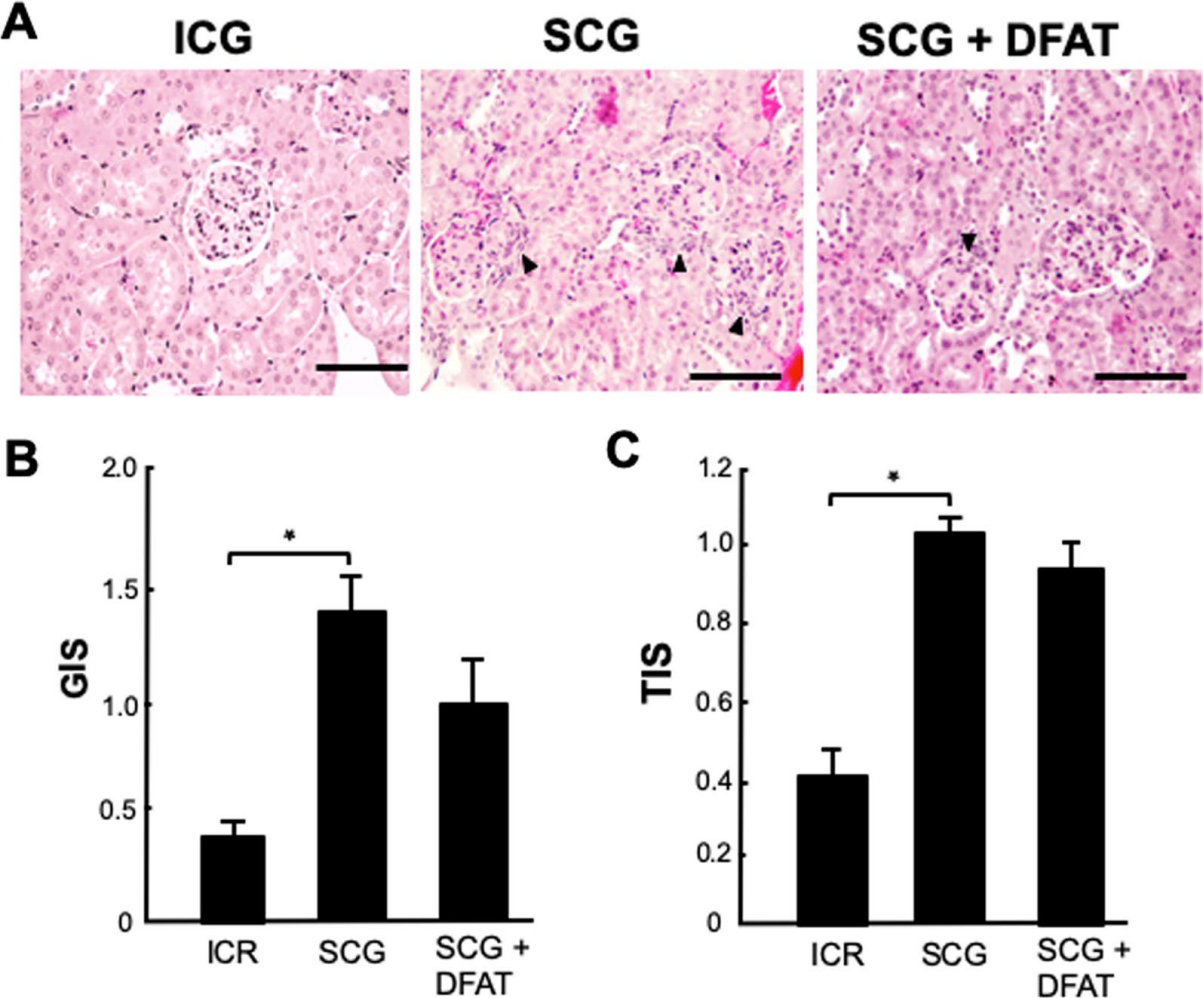
Effects of implantation of DFAT cells on renal injury in SCG mice. SCG mice were infused with 10^5^ DFAT cells through the posterior orbital venous plexus once at 8 weeks of age. Mice were killed at 12 weeks of age, and their kidneys were removed. **A** The paraffin-imbedded sections of the removed renal cortex were stained with hematoxylin and eosin. Arrowheads indicate cellular crescent formation in the glomerulus. **B** Glomerular injury score (GIS). **C** Tubulointerstitial injury score (TIS). Data are the mean ± SEM (*n* = 6). *P < 0.05 in the indicated columns. Bar = 50 μm. DFAT dedifferentiated fat; SEM standard error of the mean

The glomerulus from 12-week-old SCG mice showed cellular crescent formation (arrowheads in Fig. 4A). Implantation of DFAT cells considerably suppressed glomerular cellular crescent formation. The GIS and TIS were significantly (*P* < 0.05) higher in kidney from SCG mice than in those from ICR mice. Implantation of DFAT cells did not significantly affect the increased GIS and TIS in SCG mice (Fig. 4B, C).

### Effects of implantation of DFAT cells on urinary protein excretion in SCG mice

**Fig. 5:**
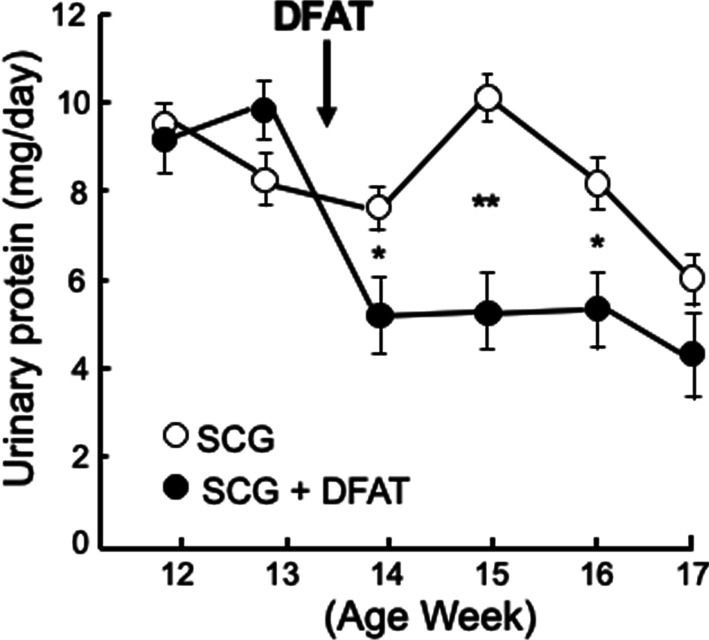
Effects of implantation of DFAT cells on urinary protein excretion in SCG mice. SCG mice were infused with 10^5^ DFAT cells through the posterior orbital venous plexus once at 8 weeks of age. Excreted urinary protein was collected from the mice each week from 12 to 17 weeks of age in metabolic cages. Data are the mean ± SEM (*n* = 6). *P < 0.05 and **P < 0.01 between with and without implantation of DFAT cells. DFAT dedifferentiated fat; SEM standard error of the mean

Next, the effects of the implantation of DFAT cells on urinary protein excretion were evaluated in SCG mice. After a single implantation of 10^5^ DFAT cells in 13-week-old SCG mice, urinary protein excretion was significantly (*P* < 0.05) lower at 14, 15 and 16 weeks of age (Fig. 5).

### Expression of TSG-6 in kidney from SCG mice after implantation of DFAT cells

**Fig. 6:**
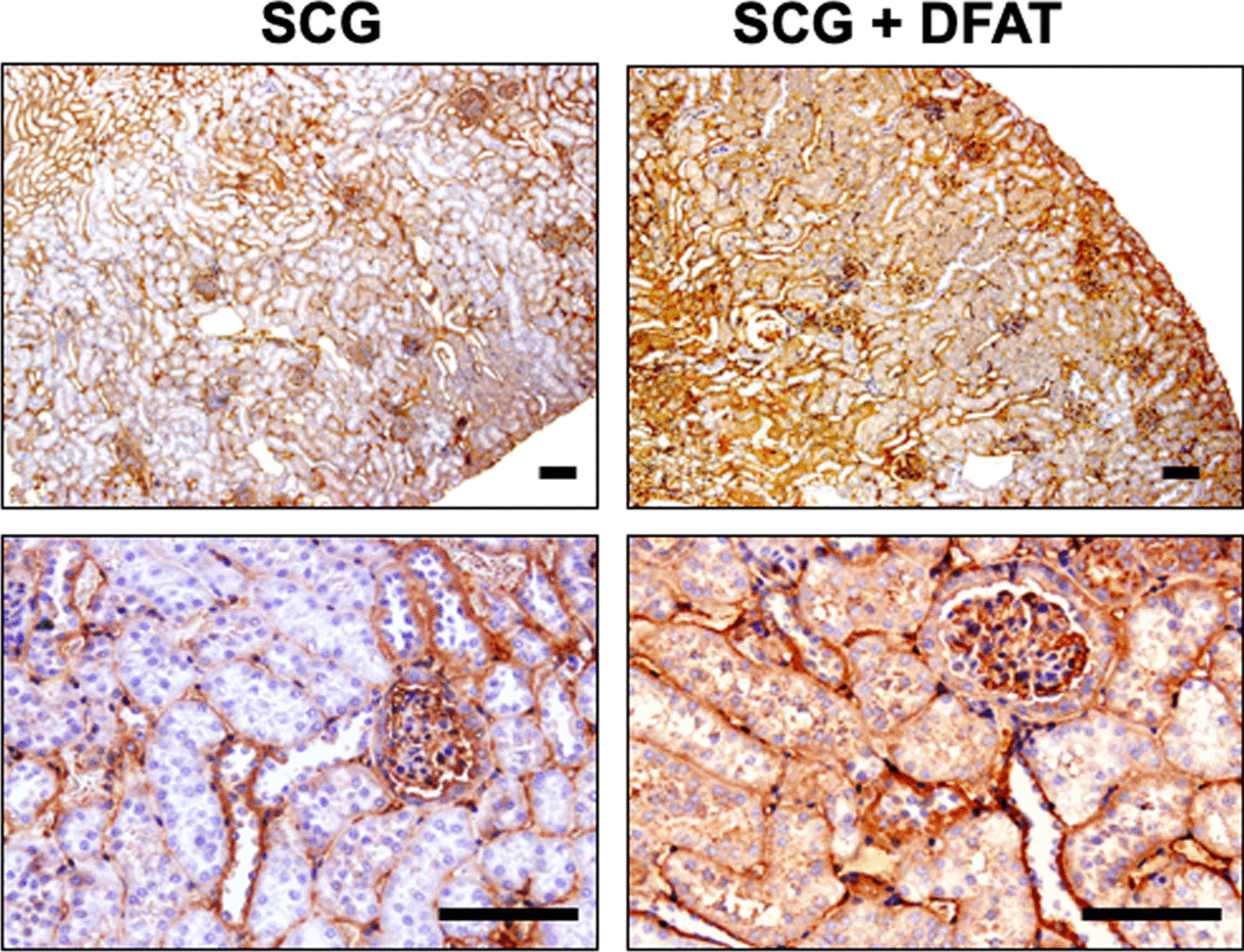
Expression of TSG-6 in kidney from SCG mice after implantation of DFAT cells. SCG mice were infused with 10^5^ DFAT cells through the posterior orbital venous plexus once at 8 weeks of age. Mice were killed at 12 weeks of age, and kidneys were removed. The 3-mm paraffin sections of the removed renal cortex were stained with rabbit polyclonal anti-TSG-6 antibody. Bar = 50 μm. DFAT dedifferentiated fat; TSG-6 tumor necrosis factor-stimulated gene-6

Effects of the implantation of DFAT cells were evaluated on immunostaining of TSG-6 in SCG mice. TSG-6 was positively stained in the glomerular mesangium but not in the nephrotubulus. After implantation of DFAT cells, immunostaining of TSG-6 was obviously increased as shown by the positive staining in the glomerular mesangium and nephrotubulus of kidney from SCG mice (Fig. 6).

### Expression of miRNA in plasma, kidney and lung

The expressions of miRNA related to TSG-6 in plasma, kidney and lung from SCG mice with or without the implantation of DFAT cells were then determined. Expression of miR23b-3p was obviously higher in plasma, kidney and lung from SCG mice with implantation of DFAT cells compared to that in plasma, kidney and lung from SCG mice without implantation of DFAT cells (Table 2).

**Table 2:** Expressions of microRNA in plasma, kidney and lung from SCG mice with or without implantation of DFAT cells

miRNA	SCG plasma	SCG plasma + DFAT	SCG kidney	SCG kidney + DFAT	SCG lung	SCG lung + DFAT
miR214-5p	2	10	202	153	210	269
miR1247-3p	27	80	20	17	18	12
miR326-5p	0	0	5	10	10	7
miR204-3p	5	7.5	44	41	14	13
miR23b-3p	643	1203.5	47,281	54,550	79,636	98,642

*DFAT* dedifferentiated fat, *SCG* spontaneous crescentic glomerulonephritis-forming

### Effects of implantation of DFAT cells on expression of immune regulator mRNAs in kidney from SCG mice

**Fig. 7:**
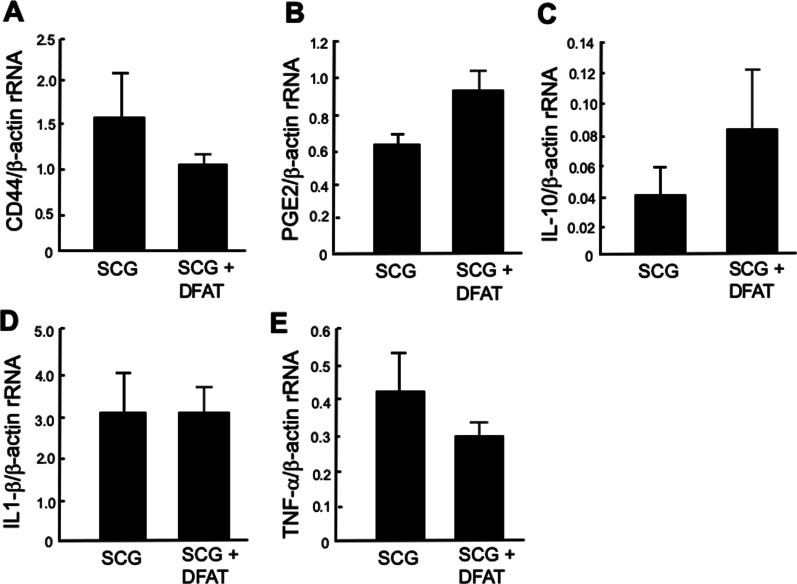
Effects of implantation of DFAT cells on the expression of immune regulator mRNAs in kidney from SCG mice. SCG mice were infused with 10^5^ DFAT cells through the posterior orbital venous plexus once at 8 weeks of age. Mice were killed at 12 weeks of age, and kidneys were removed. Total RNA was extracted from the renal medulla of kidneys with TRIzol reagent. **A** CD-44, **B** PGE2, **C** IL-10, **D** IL-1*β*, and **E** TNF-*α* and *β*-actin as an internal control were determined by SYBR Green method. Data are the mean ± SEM (*n* = 4). DFAT dedifferentiated fat; IL interleukin; PGE2 prostaglandin E2, TNF tumor necrosis factor

Implantation of DFAT cells significantly (*P* < 0.05) decreased the abundance of CD44 mRNA (Fig. 7A) and significantly (*P* < 0.05) increased that of PGE2 mRNA (Fig. 7B) in kidney from SCG mice. Implantation of DFAT cells increased the abundance of IL-10 mRNA but not statistically significantly (Fig. 7C). Implantation of DFAT cells did not affect the abundance of IL1-*β* (Fig. 7D) or TNF-*α* (Fig. 7E) mRNA in kidney from SCG mice.

### Effects of implantation of DFAT cells on expression of immune regulator proteins in kidney from SCG mice

**Fig. 8:**
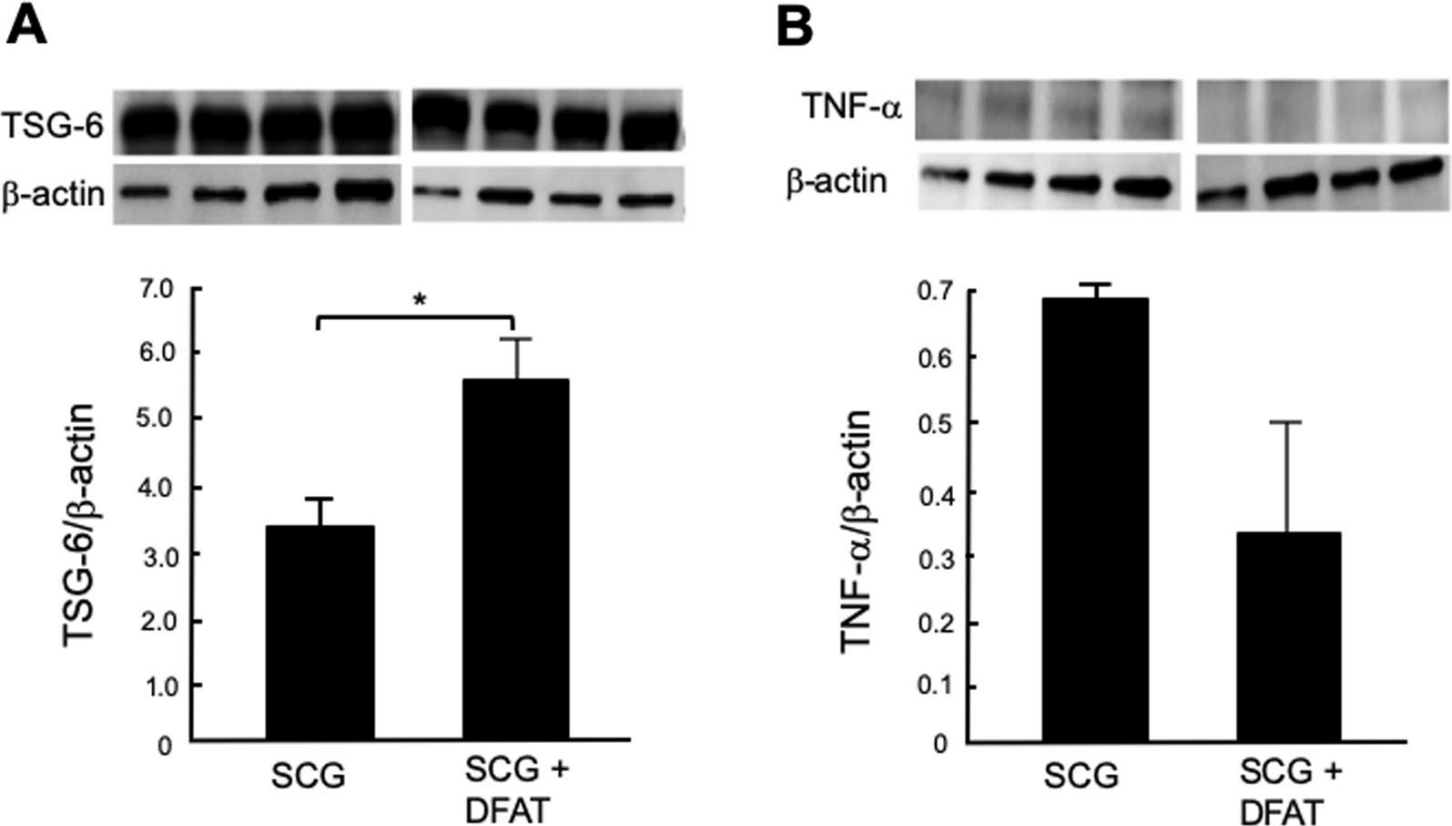
Effects of implantation of DFAT cells on expression of TSG-6 (**A**) and TNF-*α* (**B**) proteins in kidney from SCG mice. SCG mice were infused with 10^5^ DFAT cells through the posterior orbital venous plexus once at 8 weeks of age. Mice were killed at 12 weeks of age, and kidneys were removed. Protein samples were subjected to electrophoresis on polyacrylamide gels and then transblotted to nitrocellulose membranes. Blots were incubated with anti-TSG-6 polyclonal antibody and anti-TNF-*α* polyclonal antibody, and anti-beta actin antibody in TBST solution. Data are the mean ± SEM (*n* = 4). *P < 0.05 between with and without implantation of DFAT cells. DFAT dedifferentiated fat; SEM standard error of the mean; TNF tumor necrosis factor; TSG-6 tumor necrosis factor-stimulated gene-6

**Fig. 9:**
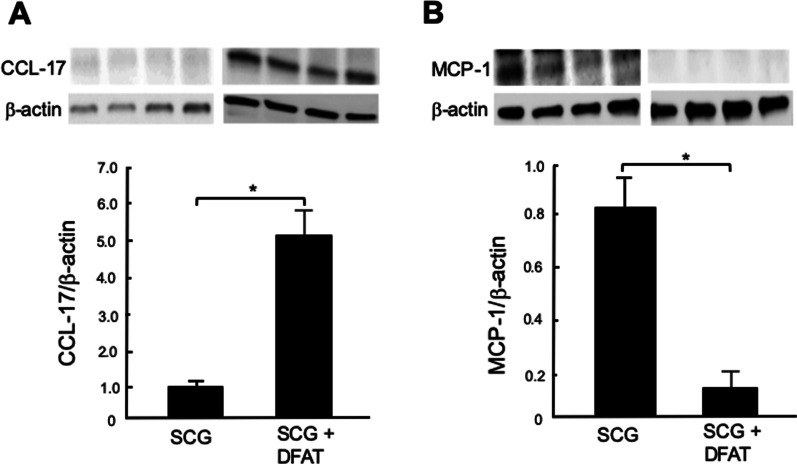
Effects of implantation of DFAT cells on expression of CCL-17 (**A**) and MCP-1 (**B**) proteins in kidney from SCG mice. SCG mice were infused with 10^5^ DFAT cells through the posterior orbital venous plexus once at 8 weeks of age. Mice were killed at 12 weeks of age, and kidneys were removed. Protein samples were subjected to electrophoresis on polyacrylamide gels and then transblotted to nitrocellulose membranes. Blots were incubated with anti-CCL-17 polyclonal antibody, anti-MCP-1 polyclonal antibody, and anti-beta actin antibody. Data are the mean ± SEM (*n* = 4). *P < 0.05. CCL-17 C–C Motif Chemokine Ligand 17; DFAT dedifferentiated fat; MCP-1 monocyte chemoattractant protein-1; SEM standard error of the mean

Implantation of DFAT cells significantly (*P* < 0.05) increased the amount of TSG-6 protein in kidney from SCG mice (Fig. 8A) but significantly (*P* < 0.05) decreased the amount of TNF-*α* protein in kidney from SCG mice (Fig. 8B). Implantation of DFAT cells also significantly (*P* < 0.05) increased the amount of CCL-17 protein, a chemokine for M2 macrophages, in kidney from SCG mice (Fig. 9A), but it significantly (*P* < 0.05) decreased the amount of MCP-1 protein, a chemokine for M1 macrophages, in kidney from these mice (Fig. 9B).

### Effects of implantation of DFAT cells on survival rate of SCG mice

Following the above, the effects of the implantation of DFAT cells on the survival rates of SCG mice with and without the implantation of DFAT cells were evaluated. Seven weeks after the implantation of DFAT cells in SCG mice, the survival rate of these mice was 89%, whereas it was 67% in SCG mice without the implantation of DFAT cells (Additional file 1: Fig. S1).

### Effects of implantation of DFAT cells on regulatory T cell population in SCG mice

**Fig. 10:**
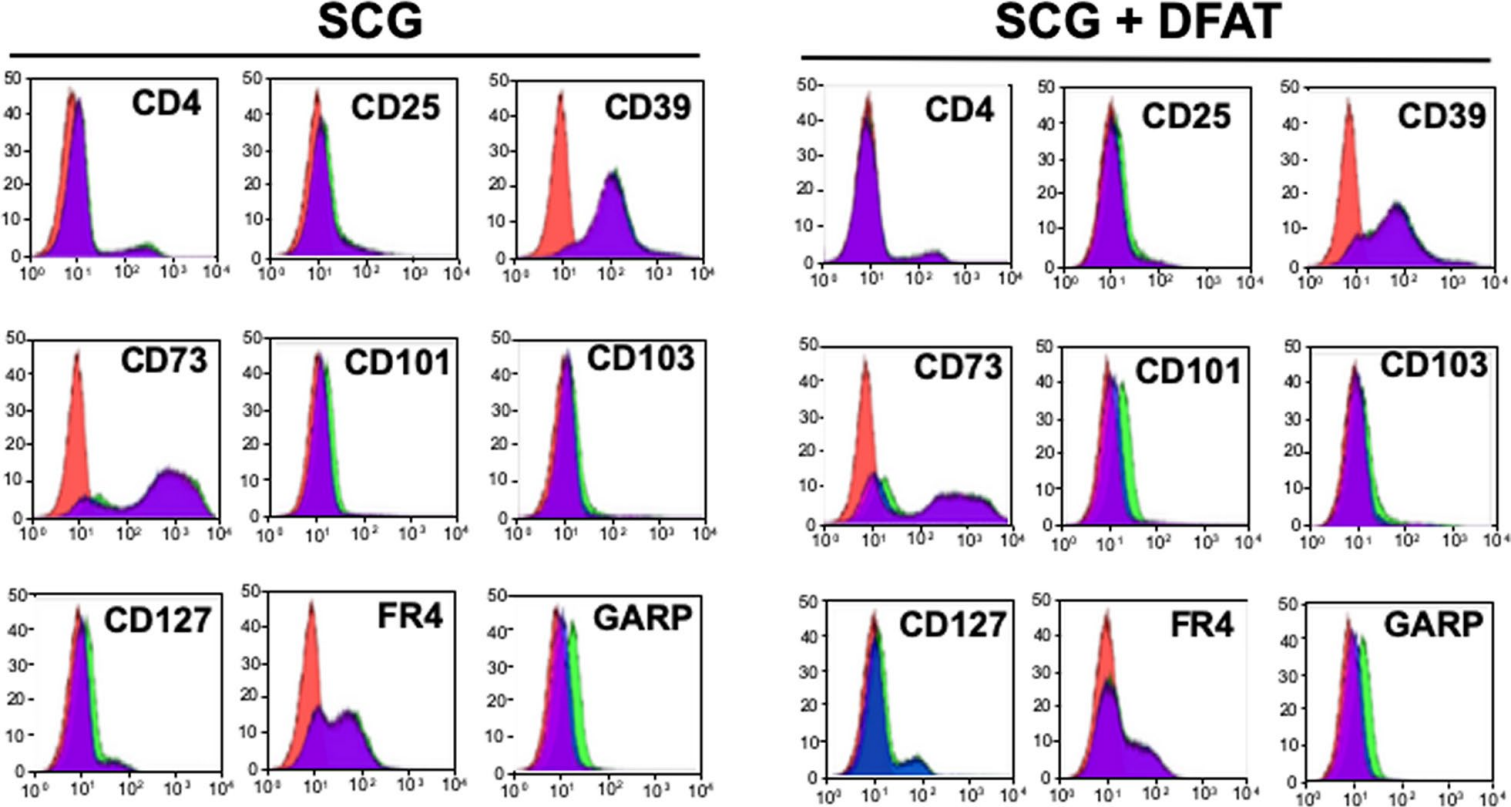
Effects of implantation of DFAT cells on the population of regulatory T cells in SCG mice. Spleen was removed and homogenized 4 weeks after implantation of 10^6^ DFAT cells in SCG mice. Cells from spleen were labeled with fluorogenic antibodies to evaluate the proportion of CD4^+^ CD25^+^ FOXP3^+^ regulatory T cells. Cells were fixed and permeabilized with a FOXP3 Staining Buffer Set according to the manufacturer’s instructions and included a blocking step with 2% rat serum. Flow cytometry was performed with a FACSAria system, and data were analyzed using FlowJo 7.6.5 software. DFAT dedifferentiated fat

Figure 10 shows flow cytometric analyses used to evaluate the proportion of CD4^+^ CD25^+^ FOXP3^+^ regulatory T cells in spleen from SCG mice with or without implantation of DFAT cells. There was no significant difference in the analyses between SCG mice with or without implantation of DFAT cells.

## Discussion

In the present experiments, the delivery of DFAT cells intravenously infused in SCG mice was first evaluated. Almost all of the DFAT cells were trapped in the lung and did not reach the kidney. Despite the non-delivery of DFAT cells into the kidney, the implantation of DFAT cells improved renal function (decrease in BUN), suppressed the expression of inflammatory cytokine MCP-1, and decreased urinary excretion of protein in SCG mice. It was previously shown that DFAT cells infused intravenously were trapped mainly in the lungs without reaching the kidneys, and implantation of DFAT cells reduced proteinuria and improved glomerulosclerosis and interstitial fibrosis in mAb 1-22-3-induced glomerulonephritis in rats. Moreover, the systematic implantation of DFAT cells through the vein was more effective in improving mAb 1-22-3-induced glomerulonephritis than direct implantation of DFAT cells through the renal artery [13]. Thus, it is surmised that the DFAT cells trapped in lungs released substrates that may have then reached the kidney to improve ANCA glomerulonephritis in the SCG mice. Mechanisms underlying the immunosuppressive effects of the MSCs trapped in the lungs after intravenous implantation have been reported to be associated with exosomes including cytokines, miRNAs and peptides that improve acute graft-versus-host disease and immune-induced acute kidney injury [18, 19]. In terms of the mechanisms underlying the induction of immunosuppressive and anti-inflammatory effects by lung-trapped MSCs and DFAT cells, these effects of the implantation of MSCs are associated with the secretion of soluble factors with paracrine actions that are mediated by exosomes. Exosomes are predominantly released from the endosomal compartment and contain miRNA, cytokines and proteins from MSCs. Recent studies in animal models suggest that exosomes have significant potential as a novel alternative to whole-cell therapies [20]. Bruno et al. [18] showed that exosomes derived from MSCs improve acute tubular injury. Gregorini et al. [21] reported that the intravenous implantation of MSCs markedly improved renal function in patient with pANCA-positive rapidly progressive glomerulonephritis, which was accompanied by normalization of urinary sediment, a significant reduction in the pANCA titer, and an increase in the regulatory T cell pool in the blood. In addition, Chaubey et al. [22] reported that implantation of MSCs improved experimental bronchopulmonary dysplasia in part via exosome-associated factor TSG-6. Thus, DFAT cell-derived exosomes may improve ANCA glomerulonephritis by increasing TSG-6 in kidney. It is thought that the direct infusion of exosomes from ex vivo-cultured DFAT cells will be effective immunosuppressive therapy for ANCA glomerulonephritis.

In the present experiments, the single implantation of DFAT cells was rather more effective in improving renal function, decreasing MPO-ANCA titer and increasing the expression of TSG-6 in the kidney of SCG mice than the triple implantations of DFAT cells. Recently, single implantation has been also reported to show rather effective immunosuppressive effects on autoimmune-induced colitis in mice [23] and osteoarthritis in a clinical study [24]. Thus, the single implantation of DFAT cells effectively induced the immunosuppression of ANCA glomerulonephritis, which may be associated with exosome release from the lungs.

In the present experiments, the ANCA glomerulonephritis showed cellular crescent formation in kidney from the 12-week-old SCG mice. Neumann et al. [25] showed that the number of glomerular crescent formations increased along with aging. In addition, plasma levels of MPO-ANCA concentrations in SCG mice were higher than those in ICR mice. Implantation of DFAT cells decreased the GIS but did not affect the TIS of kidney from ICR mice, indicating that implantation of DFAT cells mainly improved glomerular injury but did not appreciably affect nephrotubular degeneration.

Concerning the immunosuppressive effects, MCSs have been reported to release TSG-6 that suppresses adhesion molecule CD44 on T cells to inhibit T cell activity and cell infiltrations, by which the regulatory T cells are increased to obtain immune tolerance [26, 27]. It was previously observed that the systematic implantation of DFAT cells effectively ameliorated mAb 1-22-3-induced glomerulonephritis through immunosuppressive effects accompanied by the suppression of macrophage infiltration and expression of IL-6, IL-10 and IL-12*β* and increased the production of serum and renal TSG-6, which improved the mAb 1-22-3-induced renal degeneration through the immunosuppressive effects of TSG-6 [13].

The implantation of DFAT cells markedly increased the expression of TSG-6 mRNA and proteins in kidney from SCG mice, even though no DFAT cells were delivered into the kidney, but it did not affect the number of regulatory T cells in spleen from SCG mice. However, implantation of DFAT cells decreased the expression of CD44 mRNA in kidney from these mice, thus suggesting that DFAT cells may regulate immunoactivity by suppressing the activity and invasion of T cells.

Moreover, after the implantation of DFAT cells, the expression of PGE2 and IL-10 mRNAs in the present experiments was increased in kidney from SCG mice. Following their implantation, the expression of MCP-1 as M1 macrophage chemokines was decreased in kidney of SCG mice and that of CCL17 as a chemokine for M2 macrophages was increased in kidney of SCG mice. These results indicate that mechanisms underlying the immunoregulatory effects of DFAT cells appear to be associated with induction of the transition of M1–M2 macrophages with production of inflammatory cytokine IL-10. Furuhashi et al. [28] also showed that the systemic implantation of adipose-derived stromal cells protected against renal injury and decreased proteinuria, crescent formation and infiltration by glomerular leukocytes, including neutrophils, CD8^+^ T cells and CD68^+^ macrophages, in a rat model of anti-glomerular basement membrane disease. They suggested that the implantation of adipose-derived stromal cells exerts renoprotective effects in anti-glomerular basement membrane glomerulonephritis by promoting the phenotypic conversion of macrophages to immunoregulatory cells.

Because the implantation of DFAT cells may improve ANCA glomerulonephritis through increases in TSG-6 in kidney, which may be mediated by exosomes, the expressions of miRNAs in plasma, kidney and lungs were analyzed in SCG mice following the implantation of DFAT cells. The expression of miR23b-3p was obviously higher in plasma, kidney and lung from these mice with rather than without implantation of DFAT cells, thus indicating that the increases in TSG-6 in kidney from SCG mice may be mediated by miR23b-3p delivered by the exosomes.

In the present experiments, the survival rate was higher in SCG mice with rather than without the implantation of DFAT cells. Longer-term investigations of the survival rate and side effects such as tumor genesis are needed for application of the implantation of DFAT cells for ANCA glomerulonephritis as the average survival period of SCG mice is only 120 days.

## Conclusions

The intravenous implantation of DFAT cells in SCG mice suppressed renal injury including glomerular cellular crescent formation in kidney and increased the expression of TSG-6 without the delivery of DFAT cells directly into kidney. Mechanisms underlying the improvement of ANCA glomerulonephritis were associated with the immunosuppressive effects of TSG-6 and the transition of M1–M2 macrophages. These findings suggest that implantation of DFAT cells may be a potential cell therapy for ANCA glomerulonephritis.

## Supplementary Information

**Additional file 1: Fig. S1.** Comparison of survival rates in SCG mice without and with implantation of DFAT cells.

## Data Availability

The data that support the findings of this study are available from the corresponding author upon reasonable request.

## Abbreviations

ANCA: Antineutrophil cytoplasmic antibody
BUN: Blood urea nitrogen
CCL17: C–C motif chemokine ligand 17
CD: Cluster of differentiation
DFAT: Dedifferentiated fat
GIS: Glomerular injury score
IL: Interleukin
mAb: Monoclonal antibody
miRNA: MicroRNA
MCP-1: Monocyte chemoattractant protein-1
MPO: Myeloperoxidase
MSCs: Mesenchymal stem cells
PBS: Phosphate-buffered saline
SCG: Spontaneous crescentic glomerulonephritis-forming
TBST: Tris-buffered saline with 0.1% Tween 20 detergent
TIS: Tubulointerstitial injury score
TNF: Tumor necrosis factor
TSG-6: Tumor necrosis factor-stimulated gene-6
UMI: Unique Molecular Indexes
