# Supplementary material for: Implantation of dedifferentiated fat cells ameliorated antineutrophil cytoplasmic antibody glomerulonephritis by immunosuppression and increases in tumor necrosis factor-stimulated gene-6
Source: Stem Cell Res Ther. 2022 Jul 16;13:319. doi: 10.1186/s13287-022-03014-8 (PMC9288725; doi:10.1186/s13287-022-03014-8)
Supplement: Supplementary file 1 — Additional file 1: Fig. S1. Comparison of survival rates in SCG mice without and with implantation of DFAT cells. [file 13287_2022_3014_MOESM1_ESM.docx]

**SUPPLEMENTARY MATERIAL**

**Supplemental Figure 1.** Comparison of survival rates in SCG mice with and without implantation of DFAT cells. At seven weeks after the implantation of DFAT cells, the survival rate was 89% in SCG mice but was 67% in SCG mice without the implantation of DFAT cells. *DFAT* dedifferentiated fat, *iv* intravenous, *SCG* spontaneous crescentic glomerulonephritis-forming

0.0

0.2

0.4

0.6

0.8

1.0

Weeks after DFAT iv

Survival rate

4

5

6

7

8

89% (8/9)

67% (6/9)

SCG + DFAT

SCG
